# The Big Yawning: Pathological Yawning as a Symptom of Neuromyelitis Optica Spectrum Disorders

**DOI:** 10.1155/2019/9691863

**Published:** 2019-02-24

**Authors:** Veronika Spahlinger, Annette Niessen, Sebastian Rauer, Stefan Krämer, Matthias Reinhard

**Affiliations:** ^1^Department of Neurology and Clinical Neurophysiology, Medical Center Esslingen, Academic Teaching Hospital of the University of Tübingen, Tübingen, Germany; ^2^Department of Neurology and Neurophysiology, University Medical Center Freiburg, University of Freiburg, Freiburg, Germany; ^3^Department of Radiology and Nuclear Medicine, Medical Center Esslingen, Academic Teaching Hospital of the University of Tübingen, Tübingen, Germany

## Abstract

Pathological yawning is rarely observed in cerebral or spinal diseases. A 67-year-old woman was admitted with a seven-day progressive hemisyndrome with left-sided limb ataxia and hypesthesia. The patient yawned with a high frequency, partially in salve-like episodes. MRI showed a cervical myelitis over more than three vertebral segments up to the lower medulla and Aquaporin-4-antibodies were positive (diagnostic criteria for a Neuromyelitis optica spectrum disorder were fulfilled). Under treatment with methylprednisolone, followed by plasmapheresis and immunoadsorption, clinical symptoms were regressive and the frequency of yawning completely normalized. When observing pathological yawning, even in the absence of other cerebral or brainstem symptoms, one should be aware of NMOSD as a possible cause.

## 1. Introduction

Yawning is an everyday phenomenon. Pathological yawning is defined by a yawning rate higher than commonly accepted without triggers like fatigue or boredom. Various anatomical structures such as the insula, thalamus, hypothalamus, and brainstem reticular formation as well as the locus coeruleus are involved in the initiation and procedure of yawning [1]. As a symptom, pathological yawning can occur as part of epileptic seizures, premigraine, and strokes or as side effects of medications [2].

## 2. Case Presentation

**Figure 1:**
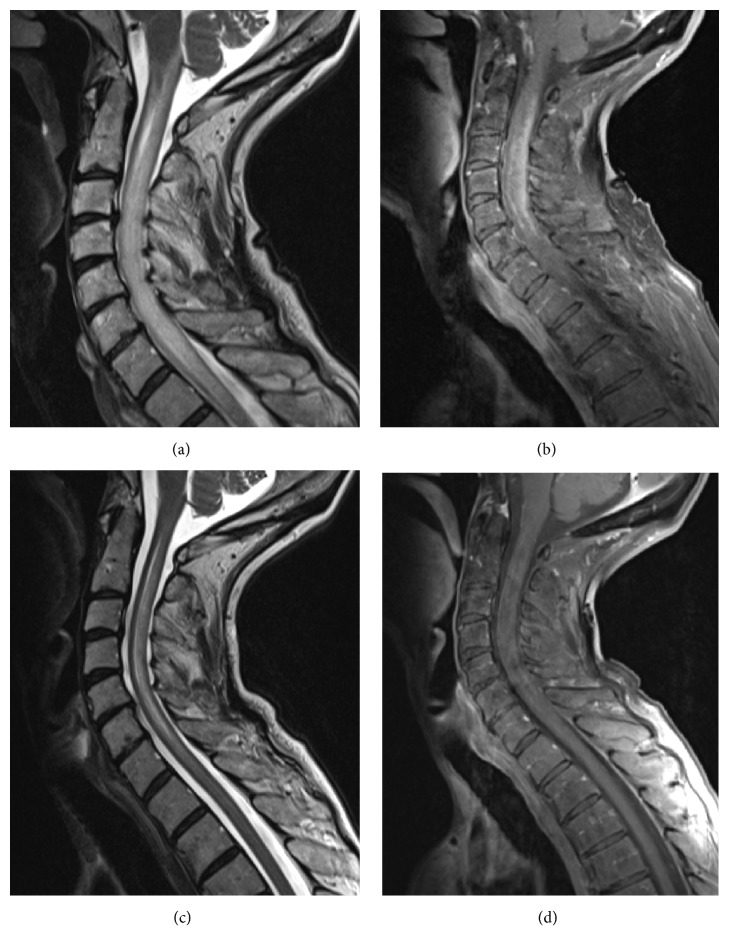
(a) Distinct oedema of the cervical spinal cord on T2-weighted sequences with spinal cord swelling and expansion into the brainstem. (b) Distinct gadolinium enhancement of the cervical spinal cord on T1-weighted sequences with cord swelling. (c) Regressive edema in the cervical spinal cord after 5 days of methylprednisolone followed by plasmapheresis and immunoadsorption. (d) Regressive gadolinium enhancement in the cervical spinal cord after therapy.

A 67-year-old woman was admitted to our hospital with a seven-day progressive hemisyndrome with left-sided limb ataxia and hypesthesia. On admission, the patient yawned with a high frequency (>3/minute) and partially in salve-like episodes (>8 yawns). There was no increased level of fatigue or boredom. Yawning was accompanied by nausea and vomiting. There were no cranial nerve deficits. The cranial MRI showed moderate leukoaraiosis not suggestive of inflammation and a signal enhancement of the coregistered myelon up to the lower medulla. The cervical MRI then revealed the full extent of the cervical myelitis over more than three vertebral segments (see Figures 1(a) and 1(b)). The cerebrospinal fluid (CSF) showed a slight pleocytosis with lymphocytes (7 cells) without elevation of CSF protein. CSF-restricted oligoclonal IgG bands were present. The visual evoked potentials were normal on both sides. The diagnostic criteria for a NMOSD were fulfilled: positive test for AQP4-IgG and acute myelitis [3]. There was no evidence for alternative diagnoses such as other systemic autoimmune, infective, vascular, neoplastic, or paraneoplastic disease. The patient had a history of autoimmune hepatitis with increased ANA titer, which was inactive under long-term treatment with low-dose azathioprine. Under initial high-dose treatment with methylprednisolone, followed by plasmapheresis and immunoadsorption, clinical symptoms of ataxia and hypesthesia and the spinal cord enhancement on MRI were regressive within 10 days (see Figures 1(c) and 1(d)). Consequently, the frequency of yawning normalized and no more salves of yawning were observed. Treatment with rituximab was initiated.

## 3. Discussion

This case shows pathological yawning as a symptom of NMOSD. So far, one series of 9 NMOSD patients with pathological yawning lasting over a period of at least two weeks was described [4]. Our case supports this previous notion of excessive yawning with NMOSD. The phenomenon of pathological yawning in NMOSD may be underrecognized by both patients and physicians because yawning is a regular physiological phenomenon. Only 2 of the previously reported 9 cases did not show other brainstem symptoms; one of them, however, exhibited hypothalamic symptoms. All these patients had abnormal brain MRI with inflammatory lesions in the hypothalamus and/or brainstem. In the present case, yawning existed only for a few days and dissolved rapidly after immune therapy. Interestingly, in our patient, no other brainstem or hypothalamic symptoms were observed apart from nausea and vomiting. MRI only showed involvement of the lower medulla including the area postrema but no other inflammatory brainstem or hypothalamic lesion. This might be due to treatment initiation prior to radiologically visible extension of the disease to more rostral structures.

Differential diagnoses for pathological yawning include ischemic stroke (in the upper brainstem, insula, or caudate nucleus [1, 5, 6], autonomic epileptic seizures [7], parkinsonism, intracranial hypertension due to infections or brain tumors, and side effects of medications (e.g., serotonin reuptake inhibitors and apomorphine) [2]. We could not find such factors in the present case as an alternative explanation.

Pathological yawning is not frequently observed in other chronic inflammatory central nervous system diseases apart from a single case report in multiple sclerosis [8]. It thus seems to be a rather characteristic clinical sign of NMOSD, which could be explained by the fact that brain regions with high aquaporin-4 expression are also those being involved in the mechanism or control of yawning. Assuming an increased local temperature with extensive NMOSD inflammation in the myelon and lower brainstem, an additional factor to the increased yawning frequency could be the thermoregulatory function of yawning via suggested brain cooling effects [9]. The fact that yawning subsided following administration of methylprednisolone, which decreases temperature of inflammatory tissue, supports this hypothesis.

In conclusion, when observing pathological yawning, even in the absence of other cerebral or brainstem symptoms, one should be aware of NMOSD as a possible cause.
